# Postoperative Serotonin Syndrome: A Case Report Highlighting Dexmedetomidine for Refractory Symptoms

**DOI:** 10.7759/cureus.79314

**Published:** 2025-02-19

**Authors:** Braden M Lopez, Won Shin, Mark D Lopez

**Affiliations:** 1 Medical Education, Creighton University School of Medicine, Phoenix, USA; 2 Emergency Medicine, Yavapai Regional Medical Center, Dignity Health, Prescott, USA

**Keywords:** autonomic instability, critical care, dexmedetomidine, postoperative complications, refractory serotonin syndrome, serotonergic drugs

## Abstract

Serotonin syndrome (SS) is a life-threatening condition caused by excessive serotonin, typically due to drugs such as selective serotonin reuptake inhibitors (SSRIs), monoamine oxidase inhibitors (MAOIs), and certain opioids. It often results from combining two serotonin-affecting drugs or excessive use of one. SS is diagnosed clinically, presenting with neuromuscular hyperactivity, autonomic instability, and altered mental status, with clonus as a key distinguishing feature. The Hunter Criteria aids diagnosis but should not be used to rule out SS. Treatment starts with discontinuing the offending agent, followed by supportive care, including airway management and stabilization of vital signs. Benzodiazepines can help control seizures and agitation, while cyproheptadine may be used in moderate to severe cases. Benzodiazepines have many side effects and cyproheptadine is only available in oral formulations, which adds complexity to effective treatment. In rare instances, SS can be refractory and may require intubation and neuromuscular paralytics. In this case, we present a case of a 42-year-old female who developed confusion, tachycardia, muscle rigidity, and hypertension after outpatient ankle surgery, where she received midazolam, fentanyl, propofol, toradol, ondansetron, meperidine, and dexamethasone perioperatively. Her daily medication also included fluoxetine, dextroamphetamine-amphetamine, and trazodone. The patient required intubation for continued diazepam administration and was admitted to the ICU. Despite improving vital signs, she remained altered with diffuse muscle rigidity. After three days of refractory SS, dexmedetomidine, an alpha-2 antagonist, was administered. The patient showed clinical improvement over the next four days and was stable enough for extubation and discontinuation of dexmedetomidine. The patient made significant clinical progress and was discharged to follow up with her primary care provider. This case highlights the importance of early detection through clinical context and not relying solely on the Hunter criteria and the potential use of dexmedetomidine as an adjuvant to benzodiazepine therapy.

## Introduction

Serotonin syndrome (SS) is a life-threatening condition that is classically characterized by the triad of mental status changes, autonomic hyperactivity, and neuromuscular abnormalities. However, SS has a wide variety of other symptoms that can be used to support a clinical diagnosis. The symptoms include spontaneous clonus, agitation, diaphoresis, tremors, hyperreflexia, fever, mydriasis, and hypertonia [1,2].

The pathophysiology of SS involves an excess of serotonin, leading to an exaggerated serotonergic response, usually caused by medication. Any serotonergic modulator can lead to SS; the most commonly implicated medications include selective serotonin reuptake inhibitors (SSRIs), serotonin-norepinephrine reuptake inhibitors (SNRIs), or monoamine oxidase inhibitors (MAOIs). SS most commonly arises from the use of multiple serotonergic agents or an overdose of a single serotonergic drug. SS is a dose-dependent phenomenon; the severity of symptoms increases with higher serotonin levels [2,3,4]. 

SS is a clinical diagnosis, as no one lab value or test will prove the presence of the condition. Measuring serum serotonin levels is a poor indicator due to the vast differences in patients’ thresholds for SS. Common tests like complete blood counts, metabolic panels, urine drug screens, head CTs, and lumbar punctures may be warranted to rule out other potential causes. Differential diagnoses include neuroleptic malignant syndrome, anticholinergic toxicity, malignant hyperthermia, meningitis/encephalopathy, and thyroid storm. SS is not a diagnosis of exclusion, the Hunter Criteria is the recommended diagnostic tool. Patients must meet one of the following criteria in the presence of a serotonergic agent [1,5,6]: 1) spontaneous clonus, 2) inducible or ocular clonus with the presence of agitation OR diaphoresis OR hypertonia AND temperature >38°C, and 3) tremor AND hyperreflexia.

Treatment of SS differs based on clinical presentation. Several key principles guide the management of SS, including the discontinuation of all serotonergic agents, supportive care, sedation with benzodiazepines, serotonin antagonists, and the resumption of necessary medications after symptom resolution. Mild cases are typically managed with supportive care, cessation of serotonergic medications, and benzodiazepines. As a patient’s symptoms progress, it is essential to prioritize autonomic stabilization, close monitoring, and using a serotonin antagonist. Patients who are critically ill present with significant hурerthermiа (>41.1°C) and oftentimes need tracheal intubation, administration of paralytics, and transfer to the intensive care unit for close monitoring [2,3,5]. 

Overall, SS is a complex, life-threatening condition that can be difficult to recognize due to the wide range of presentations. We present a case of a female in her 40s who presented with SS that did not present with clonus and was refractory to standard treatment.

## Case presentation

A 42-year-old female with a past medical history of hypothyroidism, generalized anxiety disorder, and major depressive disorder presented to the emergency room at a level IV trauma center via Emergency Medical Service from an outpatient surgery center due to agitation, tachycardia, hypertension, and muscle rigidity postoperatively.

An initial survey of the patient in the emergency room showed altered mental status, confusion, muscle rigidity, tremors, and tachycardia. GCS at presentation was 7, and the patient was maintaining her airway. Earlier that day, the patient had undergone an elective arthroscopic debridement and lateral ankle ligament repair at an outpatient orthopedic center.

The physical exam was significant for a splinted left lower extremity and rigid, hypertonic muscles resistive to passive movement and hyperreflexia. A neurological exam showed no seizure-like clonus, and the patient was noticeably confused, although non-verbal at the initial presentation. Other pertinent findings included sinus tachycardia, clear lungs on auscultation, an intact airway, and a soft, non-tender abdomen. Vital signs included a pulse rate of 136 bpm, a respiratory rate of 19 breaths/min, a blood pressure of 149/122 mmHg, 93% SpO₂, and a temperature of 36.6°C.

Upon review of surgical records, it was noted that the patient had received midazolam, fentanyl, propofol, and toradol pre- and perioperatively. In the post-anesthesia care unit, the patient had also received ondansetron, meperidine, and dexamethasone. Previous emergency room records showed that the patient had been taking albuterol, fluoxetine, dextroamphetamine-amphetamine, and trazodone.

The initial differential diagnosis was broad and included stroke, hypoglycemia, urinary tract infection, alcohol intoxication, alcohol withdrawal, delirium, metabolic disturbances, and encephalopathy. Due to the patient’s increased muscle tone, fever, and altered mental status, SS and neuroleptic malignant syndrome were both considered.

In the emergency room, a urinalysis, complete blood count (CBC) with differential, complete metabolic panel (CMP), creatine-phosphokinase (CPK), urine drug screen (UDS), and an electrocardiogram (EKG) were obtained. Diazepam and a normal saline bolus were initially given pending labs. The CBC and CMP results are shown in Tables 1-2, respectively. The CBC was significant for decreased hemoglobin and hematocrit, with a low MCV, most likely due to recent surgery. The CMP was only significant for a mildly elevated chloride. The CPK and EKG were within normal limits, and the UDS was positive for proteins, amphetamines, and benzodiazepines. The CT head showed no abnormalities.

**Table 1:** Complete blood count results

CBC	Patient lab	Standard values for females
WBC	7.4/mm^3^	4,500-11,000/mm^3^
Hemoglobin	10.8 g/dL	12.0-16.0 g/dL
Hematocrit	34.30%	36-46%
MCV	71.6 um^3^	80-100 um^3^
Platelets	346/mm^3^	150,000-400,000/mm^3^

**Table 2:** Complete metabolic panel results

Comprehensive metabolic panel tests	Patient lab	Standard values for females
Glucose	110 mg/dL	70-110 mg/dL
Sodium	136 mEq/L	136-146 mEq/L
Potassium	3.8 mEq/L	3.5-5.0 mEq/L
Chloride	107 mEq/L	95-105 mEq/L
CO2	22 mEq/L	22-28 mEq/L
BUN	13 mg/dL	7-18 mg/dL
Creatinine	0.9 mg/dL	0.6-1.2 mg/dL
Calcium	9.1 mg/dL	8.4-10.2 mg/dL

**Figure 1:**
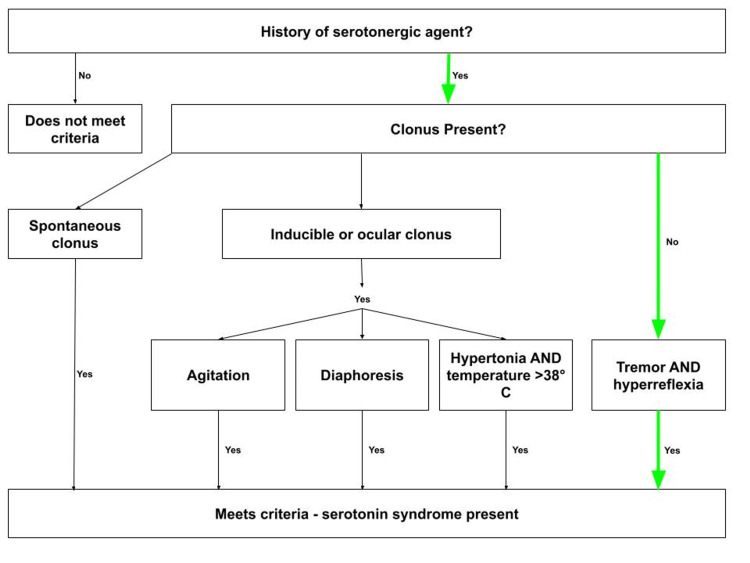
Hunter Criteria (green indicates pathway used to confirm this case's diagnosis) Authors' own creation

SS was at the top of the differential, as the patient’s physical exam, muscle rigidity with hyperreflexia, and history of serotonergic drug use matched the diagnostic criteria for SS. Poison control was contacted, and the case was discussed with a toxicologist, who agreed that SS was the most likely diagnosis. The toxicologist recommended not starting cyproheptadine but instead giving scheduled IV diazepam, as the hospital did not carry cyproheptadine and the patient could not safely swallow. The patient was admitted for overnight observation. The patient had a history of serotonergic drug use, absent clonus, but exhibited tremor and hyperreflexia on physical exam, thus meeting the Hunter Criteria, as shown in Figure 1.

While in the emergency room, the patient received a total of 40 mg of diazepam in a two-hour span, with doses of 10 mg every 30 minutes. On a repeat physical exam, the patient still had tremors with mild rigidity, but improving vital signs. However, the patient was still unable to have a coherent conversation due to altered mental status; GCS was noted to be 12. The intensive care unit (ICU) attending physician was consulted and agreed to admit the patient to the ICU for further observation.

In the ICU, the patient continued to present with confusion, inability to follow commands, tachycardia, hypertension, mild clonus, and rigidity. Due to the total amount of diazepam already administered and the need for continued dosing, the patient was intubated for airway protection. Deeper sedation was achieved with propofol, and diazepam was scheduled at 10 mg every 6 hours with periodic reassessments. The following day, diazepam was increased to every 4 hours due to persistent diffuse rigidity and unresponsiveness to verbal stimuli, especially without propofol. Tachycardia and hypertension had resolved, but due to a lack of mental status and muscle rigidity improvement, dexmedetomidine was added. It was noted that some case reports had previously described the successful use of dexmedetomidine for refractory SS [7,8,9].

**Figure 2:**
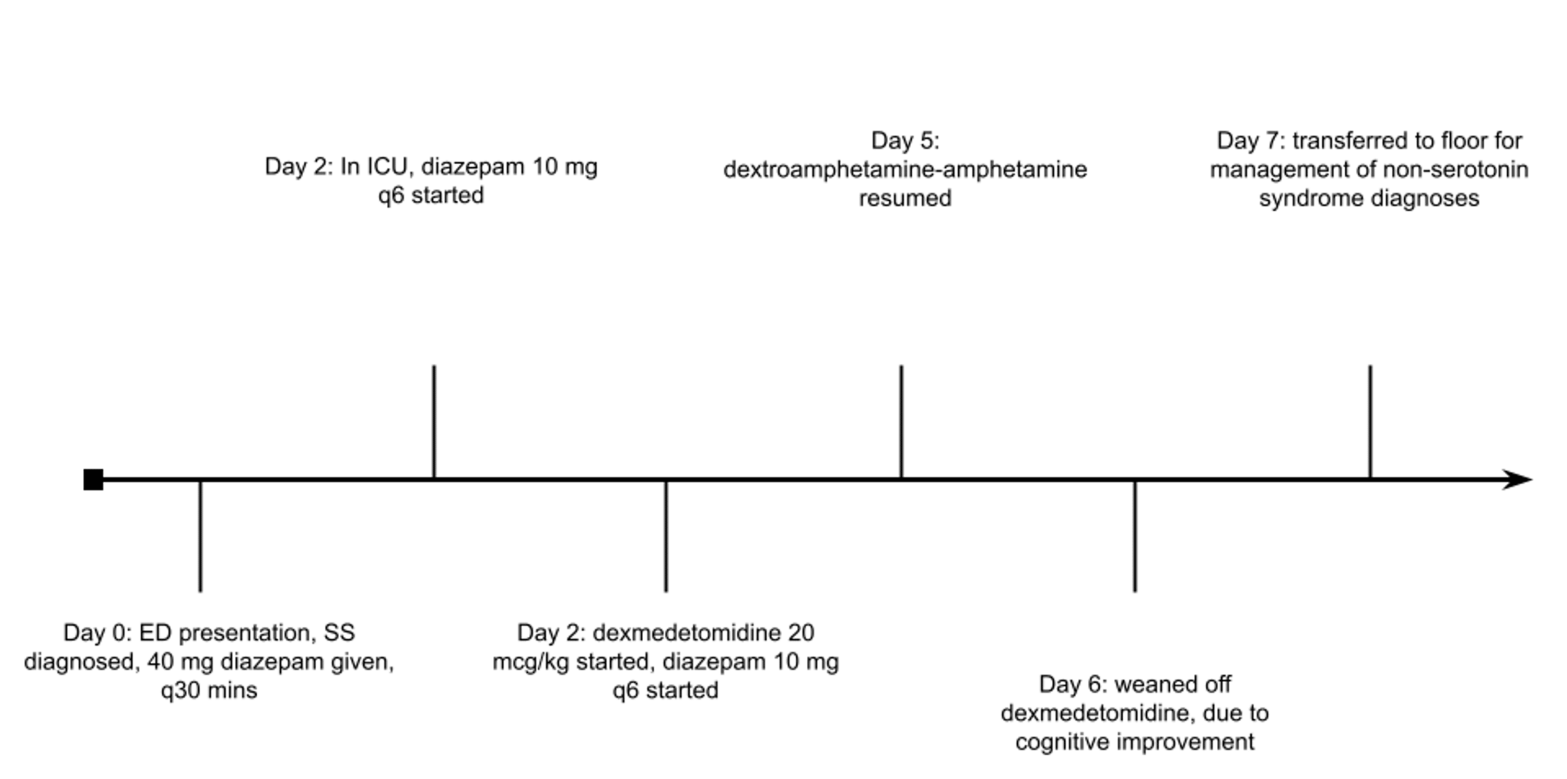
Timeline of admission

On the next day, three days after the initial presentation, the patient began to show improvement, as she became purposefully responsive. Extubation was planned, GCS was 15, and dexmedetomidine and benzodiazepines were continued at a lower dose for comfort. Dexmedetomidine was continued until day 6 of admission, when the patient was stable and near baseline. There was some intermittent rigidity daily until discharge, but the patient's vitals and mental status remained stable. The patient was medically cleared for a downgrade from the ICU on day 6, underwent an electroencephalogram (EEG) that showed no epileptiform activity and was subsequently discharged home. Figure 2 shows the patient's timeline of admission.

## Discussion

The exact incidence of perioperative SS is difficult to discern, as the current literature is limited. This is because SS is exceedingly rare. A study by Koury et al. found the incidence of SS in patients who received both fentanyl and a serotonergic agent was 0.09% [10]. One national database study done by Auerbach AD et al. showed that of 530,416 patients, over 13% of those undergoing surgery were taking an SSRI [11]. Due to the high incidence of postoperative pain in patients with psychiatric diagnoses and the relative rarity of SS, general practice guidelines favor continuing SSRIs [12]. With that being said, it is important for surgeons and anesthesiologists to collect a detailed past medical history and updated medication list. SS typically occurs secondary to serotonergic medication use, so closely monitoring any serotonergic agent, even common postoperative medications like ondansetron and fentanyl, is critical. 

Furthermore, an interesting point in this case was the use of dexmedetomidine, which led to clinical improvement. A previous case report recorded by Schlichting E et al. showed an improvement in clinical condition after dexmedetomidine administration in a 22-month-old after vilazodone overdose [9]. Kawano T et al.'s study analyzed the effectiveness of dexmedetomidine versus midazolam in rats and found that overall, dexmedetomidine was more beneficial in the treatment of SS [13]. One singular case study from Joe S et al. found the use of famotidine to prevent aspiration pneumonia in a serotonergic crisis actually decreased SS symptoms and led to stabilization [14]. There are no mentions of dexmedetomidine or famotidine on common resources like UpToDate. Further studies of dexmedetomidine versus benzodiazepines alone for the treatment of SS are indicated; however, dexmedetomidine may be effective in cases of severe refractory SS. In the majority of the literature reviewed, the mainstay of SS treatment is benzodiazepine therapy and supportive care until the symptoms reside. The use of cyproheptadine (a 5-HT2 receptor blocker) has been the long-time anecdotal treatment most discussed when treating SS but has been found to be of limited clinical use since most hospitals in the US may only carry the oral form. However, the risk of respiratory depression with benzodiazepine therapy is a definitive risk for patients, especially those unable to protect their airways. In the context of acute, life-threatening conditions, like SS, more research must be done to find an effective medication that aids in treatment without the side-effect profile of benzodiazepines.

## Conclusions

Emergency medicine providers play a pivotal role in the early recognition, initial treatment, and stabilization of SS, as evidenced in the case described. SS can present with a broad spectrum of symptoms, from mild to severe. In this instance, the patient initially exhibited altered mental status and hyperreflexia and rapidly required intubation. Notably, the patient did not exhibit any clonus, which is often a key feature in the diagnosis of SS. The patient did have a fluctuating temperature (greater than 38°C), hyperreflexia, rigidity, tremors, and altered mental status in the presence of a serotonergic agent, thus still meeting the Hunter criteria. Emergency providers must rely on clinical context and have a suspicion of SS, even if the Hunter criteria are not exactly met to ensure patients receive appropriate care. This case further serves as a point that prompt administration of treatment for SS can greatly alter the course of the condition. The administration of dexmedetomidine in this case, aided in the resolution of SS refractory to benzodiazepines, which warrants future studies to assess the potential use of the drug in treating this condition. This is one of the few case reports that demonstrate the effectiveness of dexmedetomidine in real patients experiencing a serotonergic crisis. As dexmedetomidine is not considered a validated treatment for SS, the time needed to treat is undefined. In this case, dexmedetomidine was started on hospital day 2, continued for four days, and discontinued on day 6 after mental status improved. In addition, smaller hospitals, that might not carry cyproheptadine, can use this case as a reference for the potential use of dexmedetomidine in patients experiencing severe SS.
